# Study of ultrasound-guided percutaneous microwave ablation combined with portal vein embolization for rapid future liver remnant increase of planned hepatectomy

**DOI:** 10.3389/fonc.2022.926810

**Published:** 2023-01-04

**Authors:** Qiaohong Hu, Zeng Zeng, Yuanbiao Zhang, Xiaoming Fan

**Affiliations:** ^1^ Cancer Center, Department of Ultrasound Medicine, Zhejiang Provincial People's Hospital, Affiliated People's Hospital, Hangzhou Medical College, Hangzhou, Zhejiang, China; ^2^ Department of Hepatobiliary and Pancreatic Surgery, The Second Affiliated Hospital, School of Medicine, Zhejiang University, Hangzhou, Zhejiang, China

**Keywords:** ultrasound, future liver remnant, microwave ablation, portal vein embolization, ALPPS

## Abstract

**Purpose:**

To evaluate the efficacy of ultrasound-guided percutaneous microwave ablation (PMA) combined with portal vein embolization (PVE) for planned hepatectomy.

**Methods:**

We retrospectively reviewed data of 18 patients with multiple right liver tumors or hilar tumor of liver invades the surrounding tissue and insufficient future liver remnant (FLR) for hepatectomy from July 2015 to March 2017. Ultrasound-guided PMA was performed by using PMCT cold circulation microwave treatment apparatus. PVE was performed after PMA. The increase of FLR was evaluated by computed tomography (CT) 6-22 days after PVE. The proportion of FLR, increase in the amplitude of FLR, procedure-related complications, perioperative morbidity and mortality, and overall survival (OS) rates, the median survival time were analyzed.

**Results:**

The median volume of FLR before PMA and PVE was 369.7 ml (range: 239.4-493.1 ml). After a median waiting period of 11.5 days (range: 6-22 days), the median volume of FLR was increased to 523.4 ml (range: 355.4-833.3 ml). The changes in FLR before and after PMA and PVE were statistically significant (*p*<0.001). No serious perioperative complications or mortality were found. After a median follow-up time of 51.0 months (range: 2-54 months), the 6-month, 1-year, 2-year, 3-year and 4-year survival rates were 88.9%, 72.2%, 44.4%, 33.3%, 22.2%, respectively, and the median survival time was 15.0 ± 7.1 months.

**Conclusion:**

PMA combined with PVE increases FLR rapidly, avoids touching malignant tumors, and produces fewer procedure-related complications. It appears safe and efficacious for planned hepatectomy.

## Introduction

Surgical resection is a curative therapeutic option for patients with primary or metastatic hepatic malignancies (1). Complete removal of cancer tissues by surgical resection has long been recognized to increase the survival time (2–4). However, the volume of the future liver remnant (FLR) in some patients is not enough for hepatectomy. Experienced surgeons usually consider FLR of 25% as sufficient and safe after surgical resection. And FLR in patients with hepatic dysfunction or liver injury before hepatectomy must reach approximately 40% (5–8). Recently, associating liver partition with portal vein ligation for staged hepatectomy (ALPPS) has been increasingly adopted for patients with insufficient FLR (1). The two-stage hepatectomy includes *in situ* liver splitting using laparotomy and portal vein ligation (the first stage), followed by expanded right liver hepatectomy within 7 to 10 days after the first stage (the second stage) (9). Though rapid FLR increase can be achieved, the *in situ* splitting (ISS) by laparotomy may lead to postoperative biliary leakage. Hence, we proposed using percutaneous microwave ablation (PMA) to replace laparotomy for liver partition. The study aimed to determine the effect of PMA on the liver partition.

## Patients and methods

### Patients

This study was approved by the ethical and scientific review board of Zhejiang Provincial People’s Hospital. Patients with multiple right liver tumors or hilar tumor of liver invades the surrounding tissue who were admitted to Zhejiang Provincial People’s Hospital for surgery from July 2015 to March 2017 were selected. Written informed consents were obtained from all patients. Inclusion criteria were (1) multiple tumors in the right lobe of the liver or the hilar tumor of liver invades the surrounding tissue, (2) insufficient FLR for surgery, (3) normal coagulation function and platelet count>30×10^9^/L, (4) no extrahepatic metastasis. None of the patients had received any other preoperative treatment before PMA and portal vein embolization (PVE).

### Methods

**Figure 1:**
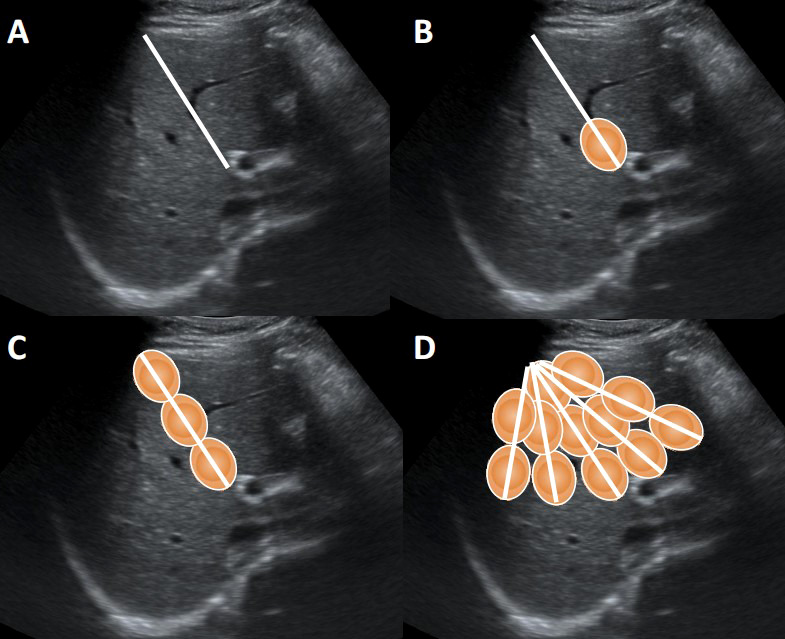
The multi-point multi-axis fan-shape ablation method. **(A)** Design needle path for ablation. **(B)** The one-point ablation. **(C)** The one-axis ablation. **(D)** The fan-shaped planar ablation.

Patients under general anesthesia received routine hepatic ultrasound examinations to determine the size and location of the liver tumors. Then ultrasound-guided PMA was performed by a PMCT cold circulation microwave treatment apparatus of computer-controlled type METI-IVD (Fuzhong Medical Hi-tech Co., Ltd, Jiangsu, People’s Republic of China), with a frequency of 2,450MHz and a maximum power of 100W. The left internal branch of portal vein and the middle branch of hepatic vein were used as the main locating vessels to determine the left and right hepatic partition surface. Under the guidance of ultrasound, percutaneous puncture was performed, and the microwave needle was placed on the determined left and right liver partition surface. According to the principle of deep first and then shallow, the multi-point multi-axis fan-shape ablation method was adopted (**Figure 1**) (10). After one-point ablation, the electrode was gradually withdrawn by about 2 cm and then microwave emission was repeated until axial ablation was formed. After one-axis ablation, the microwave needle was placed on the next axis and repeated ablated until fan-shaped planar ablation was formed. The ablation time was 2-3 minutes per point, the power was 50w, and the microwave temperature was about 90˚C. The end of the needle track was coagulated to prevent bleeding. Real-time ultrasound was used to monitor the whole process. PVE was performed after PMA. The main portal vein, left branch or right branch were punctured under ultrasound guidance, and angiography showed the position of the right portal vein before embolization. Then a guide wire was used to guide the catheter to the right portal vein branch, and embolize it with embolization coils. After that, the isobutyl cyanoacrylate (NBCA) mixture, that is NBCA and lipiodol 1:1 mixture of glue was used to have an appropriate embolization, until re-angiography showed complete embolization of the right portal vein.

### Complications evaluation

Perioperative complications were evaluated after PMA and PVE. The complications taken into consideration were high fever defined as>39.1°C, postoperative pain requiring analgesics (Grade II or higher on the World Health Organization Pain Scale), postoperative hemorrhage, and postoperative infection. To evaluate for postoperative hemorrhage, routine abdominal ultrasound was carried out 24h after operation to detect intra-abdominal hemorrhage by scanning dynamic changes in perihepatic fluid and lower abdominal fluid, combined with the dynamic change of hemoglobin (11). To assess for perioperative infection, local or systemic signs of infection (abdominal pain, fever), laboratory examination (leukocytosis), and image examination or the bacterial culture of peritoneal fluid as necessary were recorded (12).

### Efficacy evaluation

All patients underwent computed tomography and α-fetoprotein (AFP) examinations 6-22 days after PVE. The volume of FLR was measured by liver volumetric CT scan, mapping the boundary layer by layer, and then 3D volumetric imaging was performed. The volume of FLR, the proportion of FLR, increase in the amplitude of FLR, procedure-related complications, perioperative morbidity, mortality, and overall survival (OS) rates, the median survival time were recorded.

### Statistical analyses

SPSS software (SPSS for Windows 22.0, SPSS, Chicago, IL) was used for statistical analysis. The patient’s OS rate was evaluated using the Kaplan–Meier method. Stevenson’s body surface area (BSA)(cm^2^) =0.0061×height (cm) +0.0128×weight (kg) –0.1529. Standard liver volume (SLV)(ml) =706.2×BSA+2.4. FLR was calculated based on CT scan. Proportion of FLR (%) = FLR/SLV. Increase in amplitude of FLR (%) = (postoperative FLR−preoperative FLR)/preoperative FLR. In addition, the differences of FLR in patients among before and after PMA and PVE were examined by paired t-test (*** represent *p*<0.001).

## Results

### General conditions

Included in this study were 16 male patients and 2 female patients (mean age: 52.5 ± 8.3 years; range: 41.0-67.0 years). The pathological findings were assessed by two experienced pathologists independently. Of the 18 patients, 13 had hepatocellular carcinoma (HCC), 2 had cholangiocarcinoma (CC), and 3 had colorectal liver metastasis (CRLM). According to liver function Child-Pugh grading, 16 patients were in grade A and 2 in grade B. The diameter of the tumors was 81.8 ± 39.7mm (range:30.0-155.0mm). No procedure-related complications were found in our patients. The general conditions of the patients were presented in **Table 1**.

**Table 1:** Characteristics of patients.

Characteristics	Number of patients
Sex	
Male	16
Female	2
Age (years)	
Mean	52.5 ± 8.3
Range	41.0-67.0
Size of tumor (mm)	
Mean (mean ± SD)	81.8 ± 39.7
Range	30.0-155.0
Child-Pugh class	
A	16
B	2
C	0
Hepatitis history	
HBV	14
HCV	1
None	3
Liver fibrotic status	
Yes	12
No	6
Pathology	
HCC	13
CC	2
CRLM	3

### Increase of FLR

**Figure 2:**
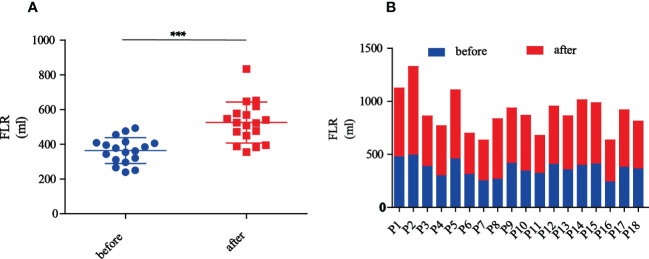
The changes of FLR before and after PMA and PVE. **(A)** The overview of FLR changes in all patients before and after PMA and PVE (*** represent p<0.001). **(B)** Each patient’s FLR changes before and after PMA and PVE.

**Figure 3:**
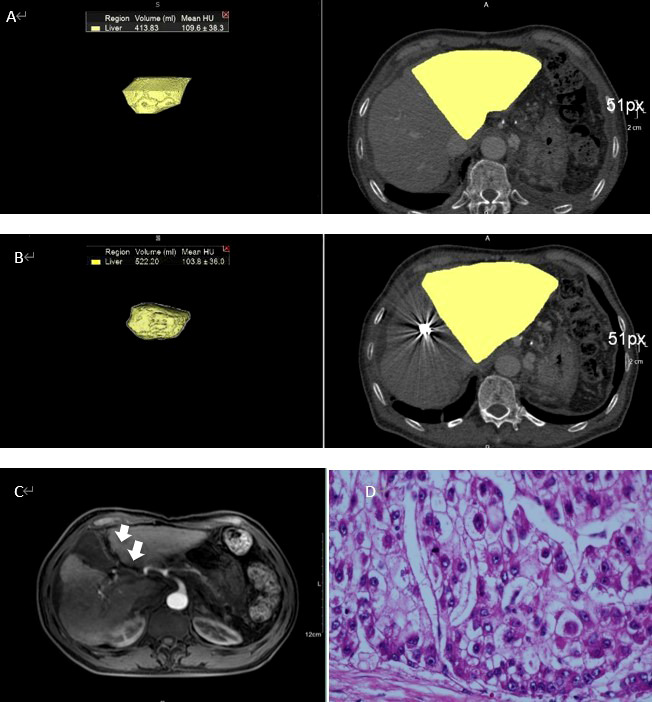
A 65-year-old man with HCC treated with PMA combined with PVE **(A)** Volume of left lobe of liver was 413.83ml according to CT. **(B)** Ten days after PMA and PVE, volume of left lobe of liver increased to 522.20ml. **(C)** The plane of PMA could be clearly seen (white arrow). **(D)** The histologic diagnosis was HCC.

Preoperative CT volumetry of the left lateral lobe showed a median volume of 369.7 ml (range: 239.4-493.1 ml). After a median waiting period of 11.5 days (range: 6-22 days), the median volume of the left lateral lobe increased to 523.4 ml (range: 355.4-833.3 ml). The median proportion of FLR was 33.4% preoperatively, which was increased to 45.0% postoperatively. The mean increase in the amplitude of FLR was 45.6% and the largest was 114.3%. The changes in FLR before and after PMA and PVE were statistically significant (*p*<0.001) (**Figure 2**). **Figure 3** showed the detailed data of a 65-year-old patient treated with PMA combined with PVE. Before the procedure, the volume of his left lobe was 413.83 ml based on a CT scan. Ten days after PMA and PVE, the volume of his left lobe reached 522.20 ml. The plane of PMA (white arrow) could be seen clearly. The histologic diagnosis of HCC was made in this patient.

### Complications

Of the 18 patients, 11 suffered low fever (≤39.1) and 9 suffered mild pain that didn’t require analgesics, but no high fever and severe pain were found after PMA and PVE. Abdominal ultrasound was carried out 24h after the operation to monitor intra-abdominal hemorrhage, and no dynamic increase of perihepatic fluid or lower abdominal fluid was found. No dynamic decrease in hemoglobin. According to the local or systemic signs, laboratory examination, image examination and the bacterial culture of peritoneal fluid if necessary, no perioperative infection was found. In addition, transaminase was temporarily elevated in some patients after the operation and decreased before the second step of planned hepatectomy due to liver protection therapy.

### Overall survival rates

**Figure 4:**
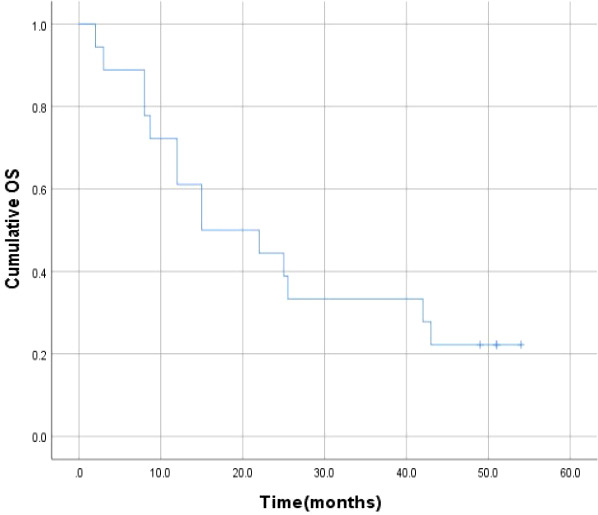
Cumulative OS for all patients.

After a median follow-up time of 51.0 months (range: 2-54 months), the 6-month, 1-year, 2-year, 3-year and 4-year survival rates were 88.9%, 72.2%, 44.4%, 33.3%, 22.2%, respectively, and the median survival time was 15.0 ± 7.1 months (**Figure 4**).

## Discussion

Due to the high incidence of hepatitis B virus (HBV) infection, the incidence of HCC in China is high (13–15). The safety of liver resection, the most effective way to treat HCC, mainly depends on FLR volume. Inadequate FLR volume is associated with postoperative liver failure, especially for patients with liver cirrhosis. In order to address the problem of insufficient FLR volume, Schnitzbauer et al. in 2012 presented ALPPS, including initial surgical exploration, right portal vein ligation, and *in situ* splitting (ISS) along the falciform ligament of the right side, for staged hepatectomy (1). ALPPS could induce 75% rapid hypertrophy of the left lateral lobe in patients after a median waiting period of 9 days. Though it can bring a higher chance of complete tumor resection, ALPPS, which includes 2 operations within a short period, may result in a higher risk of serious complications (16). Some surgeons doubted the safety of ALPPS and had to weigh the benefits of complete resection against the risk of complications. Schnitzbauer et al. reported that 24% of patients suffered from bile leakage and the mortality was 12% (1). Considering that bile leakage might be caused by ISS, Robles Campos et al. modified this procedure using a round liver ligation to replace ISS and thus avoid severe bile leakage (17). Gringeri et al. presented a new minimally invasive laparoscopic microwave ablation and portal vein ligation for staged hepatectomy (LAPS) (18). LAPS showed a relatively lower hypertrophic rate (48.7%) compared with ALPPS (74%), and it imposed enormous stress upon patients, especially for HCC patients with liver cirrhosis, because they had to endure two laparotomies (19).

In our study, we used PMA to replace ISS. In recent years, thermal ablation including radio-frequency ablation (RFA) and PMA has shown great potential in treating liver tumors. Seki et al. first reported PMA and then used it extensively in China and Japan (20). Microwave ablation is influenced by the blood supply of the tumor (21, 22). When the tumor is close to the large vessels, the rich blood supply carries away the heat of ablation and microwave ablation is simply less susceptible to the “heat-sink” effect when performed adjacent to large vessels compared to radiofrequency ablation (23–25). Compared with RFA, PMA has several advantages, including a larger ablation area in a shorter time, less susceptibility to the impact of heat sinks or perfusion, and no requirement of ground pads (26–28). Fever and pain are common complications after PMA. During PMA, apoptotic pathways are triggered, and inflammatory mediators are released into the blood at high levels (29). When the tumor is near the liver capsule or at the lower part of the diaphragm, PMA may cause liver capsule tension, coagulation, necrosis, hyperemia, and edema, thus leading to pain. But both fever and pain can be relieved by symptomatic treatment. Previous studies have also reported that microwave ablation may cause some serious complications such as hemorrhage and bile fistula (30, 31). Yet, these life-threatening complications did not occur in our patients.

The reasons why we used PMA were as follows. 1) Compared with RFA, PMA, which uses the microwave to heat water molecules, can create a larger and more homogeneous ablation area (32–34). 2) Having multiple antennae, PMA ablates a wider zone and can be performed quickly (33–35). 3) Since PMA is performed at a stable temperature and does not require grounding pads, which helps to protect the skin as much as possible from burning. However, some researchers expressed concern over PMA about its safety, short-term efficacy, long-term clinical outcomes, and prognosis in the treatment of HCC. Hong et al. reported a case of a 41-year-old male patient who was diagnosed with a liver mass by ultrasound and received PMA for liver partition and subsequent PVE for liver hypertrophy (36). Therefore, we conducted this retrospective study to evaluate the value of noninvasive PMA in liver partition and to prove whether it combined with portal vein embolization could increase FLR rapidly and safe for the second step of planned hepatectomy by analyzing the procedure-related complications, perioperative morbidity and mortality, and overall survival rates.

Schnitzbauer et al. presented that the first step of ALLPS induced rapid hypertrophy (about 75%) of the left lateral lobe in patients after a median waiting period of 9 days (1). Erik et al. reported 80% hypertrophy after a median waiting period of 7 days, almost doubling the remnant in a short period (37). Andrea et al. found rapid liver hypertrophy after ALPPS step I in mice, suggesting the contribution of the IL-6-TNF-α-STAT3 pathway (38). Our study presented similar results. After PMA and PVE, the median volume of the left lateral lobe increased from 369.7 ml to 523.4 ml, with a mean increase of 45.6% within a waiting period of 11.5 days (range: 6-22 days). Among all, whose FLR increased from 265.3 ml to 568.5 ml in a 49-year-old male patient, with a 114.3% increase in amplitude in a 15-day waiting period.

We were surprised to find that after PMA and PVE, three patients’ tumors were significantly smaller than before. The decrease in tumor size may be induced by the division of liver parenchyma between the right and left liver lobes during the procedure.

Previous studies found standard liver resection had perioperative mortality of up to 3% (39, 40). The mortality of complex liver resection was likely to be 5%-8% (41–43). Serious bile leakage, and sometimes even hepatic failure, may occur during the ISS of ALPPS. The perioperative mortality of ALPPS is higher, about 12% to 28% (1, 16, 44, 45). In our study, PMA replaced ISS with laparotomy as the first step of ALPPS. The blood of the portal vein between the tumor lobe and other lobes was split. There was no serious perioperative mortality in our study population. Besides, the 6-month, 1-year, 2-year, 3-year and 4-year survival rates were 88.9%, 72.2%, 44.4%, 33.3%, 22.2%, respectively, and the median survival time was 15.0 ± 7.1 months.

Multiple factors can affect the prognosis of PMA+PVE. The first factor is the Child-Pugh class. We consider a better prognosis is correlated with a better Child-Pugh class. Among the 18 patients, two were in Child-Pugh class B and the others were in class A. In the third month, intrahepatic metastasis or peritoneum metastasis was found in these two Child-Pugh class B patients, who then died in the third and eighth months. The second factor affecting the prognosis of surgery is age. Older patients could hardly tolerate invasive surgery twice in a short period. However, no statistically significant difference was found in survival rates between patients older than 60 and those younger than 60 in our study. This may be related to the small sample size of our study or the condition of the patients.

The limitations of our study were as follows: First, the study was completed in one center (Zhejiang Provincial People’s Hospital) and the number of patients was relatively small. Second, although some reports defined bilirubin lower than 50μmol/L as a suitable condition for liver resection, there was still no standard for preoperative biliary drainage. Third, this study did not include a comparable control group with similar characteristics such as FLR and tumor size. This limitation is inherent in our study because of the novelty of the PMA+PVE procedure. Despite these limitations, our study currently presented desirable results, suggesting that PMA combined with PVE increases FLR rapidly and appears safe and efficacious for planned hepatectomy. To verify our results, we will enlarge the sample size and record the survival situation of patients in our future research.

## Data availability statement

The original contributions presented in the study are included in the article/supplementary material. Further inquiries can be directed to the corresponding author.

## Ethics statement

This study was approved by the ethical and scientific review board of Zhejiang Provincial People’s Hospital (2016KY141). Written informed consent for participation was not required for this study in accordance with the national legislation and the institutional requirements.

## Author contributions

Conception and design of the study: XF and QH. Acquisition of data: XF, QH and YZ. Analysis and interpretation of data: QH and ZZ. Revising the article critically for important intellectual content: XF and QH. Drafting the article: QH and ZZ. All authors contributed to the article and approved the submitted version.
